# Study on Development of Composite Hydrogels With Tunable Structures and Properties for Tumor-on-a-Chip Research

**DOI:** 10.3389/fbioe.2020.611796

**Published:** 2020-12-22

**Authors:** Zaozao Chen, Fei Wang, Jie Zhang, Xiaowei Sun, Yuchuan Yan, Yan Wang, Jun Ouyang, Jing Zhang, Tess Honore, Jianjun Ge, Zhongze Gu

**Affiliations:** ^1^State Key Laboratory of Bioelectronics, School of Biological Science and Medical Engineering, Southeast University, Nanjing, China; ^2^Institute of Biomaterials and Medical Devices, Southeast University, Suzhou, China

**Keywords:** hydrogel, 3D-ECM, microchip, bio-printing, cancer invasiveness

## Abstract

A major factor for developing new tumor models is to recreate a proper three-dimensional environment for 3D tumors culture. In this 3D microenvironment, extracellular matrices play important roles in regulation of hallmark features of cancer through biochemical and mechanical signals. The fabrication of a mechanical and biophysical controllable hydrogel, while sharing similarities with Matrigel in cancer invasiveness evaluation, is an urgent but unmet need. In this study, we developed a hybrid hydrogel system composed of GelMA and hydrolyzed collagen to model tumor micro-environment and tested with several cancer cells with different origin and characteristics. This hydrogel possesses a well-ordered homogenous microstructure, excellent permeability and an adjustable mechanical stiffness. This hydrogel demonstrated similar properties as Matrigel in tumor spheroids culture and 3D tumor invasiveness studies. It was further applied in a Tumor-on-a-Chip system with 3D-bioprinting. Our research demonstrated this hydrogel's effectiveness in tumor 3D culture, and its potential to replace Matrigel in cancer invasiveness evaluation.

## Introduction

In cancer research, *in vitro* cell based models have been widely used for the purpose of examining signaling pathways and mechanisms which are responsible for different phenotypes and functions of cancer cells. Some of these include, metabolism, growth, migration, matrix invasion, and drug resistance (Sharma et al., 2010; Goodspeed et al., 2016). Conventionally, monolayer cultures of adherent cancer cells have been used for such applications (Sharma et al., 2010; Ham et al., 2016). The ease of creation and maintenance of 2D cultures of cells, and their compatibility with a number of culture vessels and biochemical assays, makes 2D cultures vital to cancer research (Hughes et al., 2011). However, despite these advantages, 2D cultures fail to recapitulate the pathophysiological features of human tumors as shown by a growing understanding of the complexity of cancer. Because of the need for improved *in vitro* cancer models, intense research on the subject matter has been done in both industry and academia. This has resulted in the development of multiple 3D culturing systems (e.g., Tumor -on-a-Chip) which serve as high fidelity tools for basic cancer research and for drug discovery applications (Weigelt et al., 2014). 3D models allow for the exploration of a wide range of variables that affect tumor growth, invasion, and metastasis. Furthermore, they provide the capability of high-throughput drug-screening that is not possible with *in vivo* animal models or clinical samples (Li and Kumacheva, 2018).

The 3D microenvironments for cancer spheroid growth is very important. Hydrogels, reconstituted by extracellular matrix (ECM) components, are often used to create 3D environments for *in vitro* studies. This is possible because of their ability to mimic the natural bioactivity of physiological environments. Natural and synthetic biomaterials and their mixtures have been increasingly utilized to develop *in vitro* models to study various cancer cell behaviors in 3D microenvironments. Protein-based hydrogels such as collagen (Szot et al., 2011; Charoen et al., 2014; Jeong et al., 2016), Matrigel (Weaver et al., 1997; Lang et al., 2001; Härmä et al., 2010; Chaudhuri et al., 2014), or fibrin (Liu et al., 2012) are commonly used for 3D cancer cell culture owing to their specific chemical and biophysical properties, within which, Matrigel is recognized as a “golden standard” scaffold *in vitro*. Matrigel is a basement membrane (BM) extract composed of a complex mixture of over 1,000 proteins, including type IV collagen, laminin and nidogen as major components (Kratochvil et al., 2019). As the most commonly-used material for 3D cell and organoid culture, Matrigel ultimately augments the self-assembling capacity of PSCs (Kleinman and Martin, 2005; Hughes et al., 2010). The applications of Matrigel, have greatly exceeded other biomaterials since its initial development several decades ago. This is attributed to several major advantages which include: built-in complex distribution of nutrients and protein gradients, ease of handling, fast gelling kinetics, and the readily available commercialized product with high quality control. Apart from this however, Matrigel possesses several notable limitations in the area of tissue engineering. Firstly, the inherent compositional variability usually results in a lack of control over individual specific microenvironmental parameters. More importantly, due to a combination of growth factors in Matrigel, the simultaneous occurrence of signaling cascades may confound signal transduction in cells undergoing organogenesis. This may lead to an incomplete understanding of self-assembly mechanisms (Vukicevic et al., 1992). Secondly, precise control over gelation kinetics is not possible due to the rapid gelling of Matrigel. This creates uncertainty in the microstructure of the final network (Cruz-Acuña and García, 2017). In addition to this, the inability to manipulate or control its mechanical properties, restricts its application in mechanotransduction during organogenesis. Lastly, although Matrigel is a widely supplied commercialized product, problems with reproducibility could still arise. This is due to inherently inconsistent composition and batch-to-batch variability. Problems with consistency can lead to problems with genetic drift in organoid formation, which is particularly important if investigators subculture and passage organoids. Therefore, biomaterials with defined component content and adjustable mechanical are in demand to replace Matrigel for organoid culture (Thakuri et al., 2018; Kratochvil et al., 2019).

Physical properties of tumor environments regulate migration and invasion of cancer cells *in vivo* (Hynes, 2009; Schedin and Keely, 2011). However, most commonly used biomaterials for 3D models do not allow flexible control of mechanical and biophysical features (Szot et al., 2011). These effects on the prometastatic functions of cancer cells can be reproduced *in vitro*. Here, several studies demonstrate that the mechanical rigidity of a hydrogel regulates aggressive behaviors of cancer cells and tumor spheroids (Ulrich et al., 2009; Liang et al., 2011; Schedin and Keely, 2011; Taubenberger et al., 2016). Changing stiffness of a hydrogel is accompanied by alterations in gel microstructure, and the consequent changes in 3D permeability affect cell viability (Cha et al., 2010).

Biopolymer hydrogels offer a wide assortment of biochemical and biophysical properties for cell morphogenesis and function. However, factors such as the range of cues provided by natural scaffolds, their batch-to-batch variation, and uncontrolled degradation, often limit the isolation of the effect of a specific ECM property on cancer cell fate. Moreover, it can affect reproducibility of the results of comparative studies of cancer cell growth (Benton et al., 2014). These limitations may be overcome with the use of synthetic hydrogel scaffolds—which carry appropriate cell adhesion ligands and biodegradable cross linkers that control hydrogel composition and properties (Sung et al., 2011; Kratochvil et al., 2019).

Gelatin-methacrylate (GelMA) has recently emerged as an attractive option for fabricating engineered ECM-based matrices, since it possesses a wide range of physical properties while maintaining constant gelatin concentration (Chen et al., 2012). In this paper, we reported generation of a simple and effective composite hydrogel with tunable structures and properties. This approach also permits the manipulation of scaffold stiffness without changing collagen content, by changing the ratio of GelMA to collagen of scaffold stiffness without changing the collagen content. Specifically, we compared the invasion and growth of invasive MDA-MB-231 breast cancer cells and NCI-H23 lung cancer cells in this composite hydrogel with non-invasive cancer cells such as MCF-7 breast cancer cells and HT-29 colon cancer cells. This GelMA-based hydrogel demonstrated its ability to simulate the functions of Matrigel in support of 3D cancer growth, measurement of cancer invasiveness, and evaluation of drug sensitivity. In addition, it exceeded the Matrigel in its ability for its usage in 3D-bioprinting and Tumor-on-a-Chip fabrication.

## Materials and Methods

### Materials and Cells

MDA-MB-231 and MCF7 human breast cancer cell lines, HT-29 human colon cancer cell lines, and NCI-H23 human lung cancer cell lines, were purchased from the Shanghai Institutes for Biological Sciences, Chinese Academy of Sciences (ACS). Leibovitz's L-15, high-glucose Dulbecco's Modified Eagle's Medium (DMEM), McCoy's 5A (Modified), and RPMI 1640 media, fetal bovine serum (FBS), Insulin-Transferrin-Selenium-A Supplement and Penicillin-Streptomycin Solution were from GibcoTM. All chemicals were purchased from Sigma-Aldrich.

### Hydrogel Formation and Optimization

**Figure 1:**
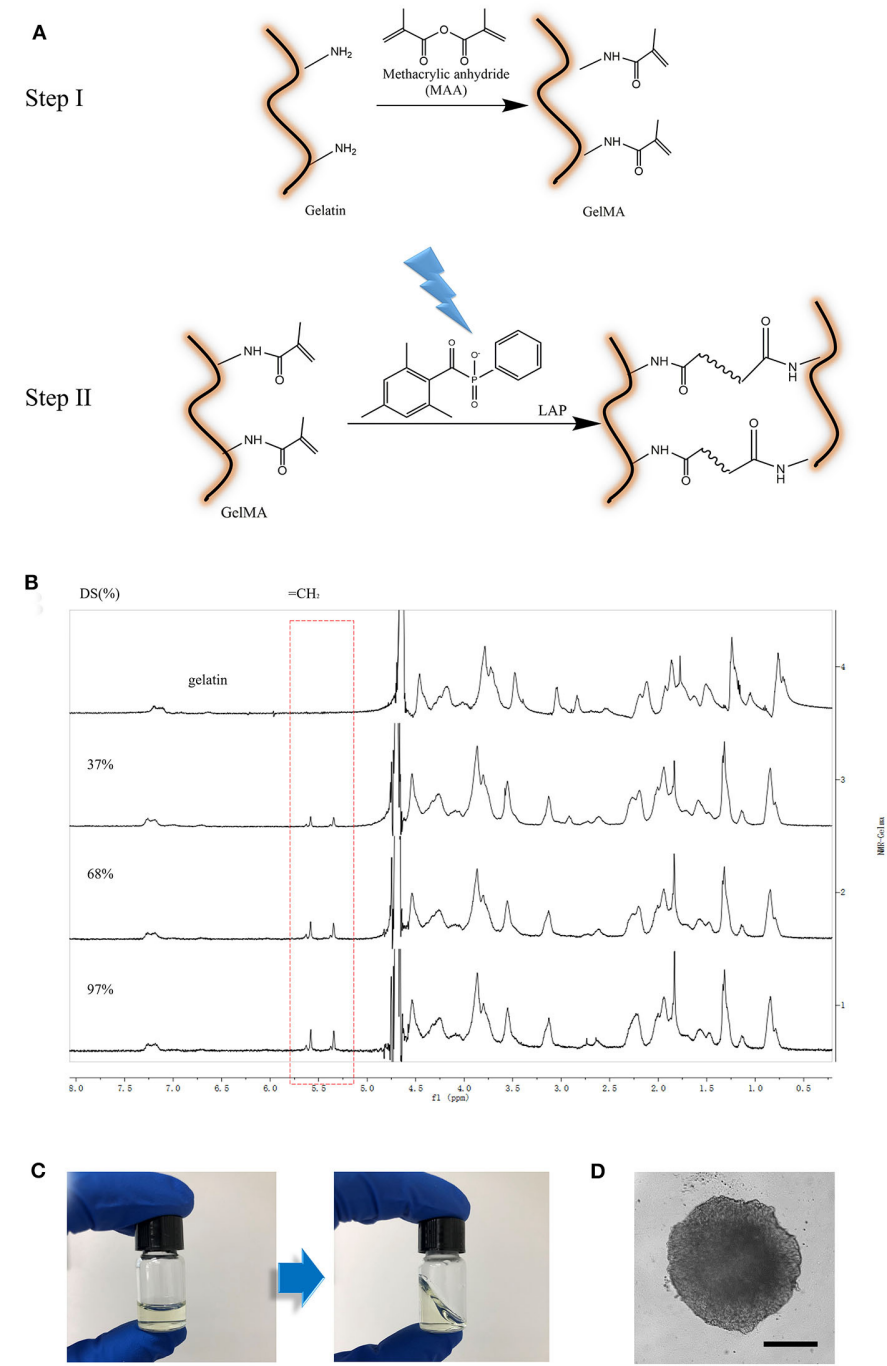
Preparation of GelMA material. **(A)** Schematic illustration of GelMA reaction; **(B)** ^1^H NMR verification of GelMA. Peak correspond to acrylic protons (2H) of methacryloylated grafts of lysine groups. **(C)** GelMA gelation (L-G) with 0.5% w/v LAP cross-linked with 405 nm light for 1 min. **(D)** image of 3D culturing of cancer Spheroids inside of crosslinked L-G. Scale Bar, 200 μm.

GelMA was synthesized using previously published procedure with some modifications (Chen et al., 2012) (Figure 1A). Briefly, porcine skin gelatin was dissolved at 10% w/v in phosphate buffered saline (PBS) at 50°C. Methacrylic anhydride (MA) was added to the gelatin solution using a peristaltic pump at a rate of 20 ml/min under aggressive stirring. Final MA concentrations of 3, 5, and 10% v/v were used and will be referred to as L-G, M-G, and H-G herein. The reaction proceeded for 24 h at 50°C shielded from light. The solution was ultrafiltrated to remove unreacted MA. The GelMA solution was filtered, lyophilized, and stored at −20°C.

To generate the GelMA-based composite hydrogels, GelMA was first dissolved at 20% w/v in PBS and incubated in a 50°C water bath until dissolved. The different degree of substitution GelMA solutions were blended in same ratios to tune the gel mechanical properties, with the goal of producing three different gel stiffness conditions. The GelMA solution was then combined with the photo-initiator lithium phenyl-2,4,6- trimethylbenzoylphosphinate (LAP; 0.05% w/v final concentration) (Fairbanks et al., 2009), PBS, and collagen in a 37°C water bath. For SEM, elastic moduli testing and culturing cancer cells and tumor spheroids, we prepared gels with same concentration (5% w/v) of different DS GelMA (L-G,M-G and H-G) and the two different collagen concentrations at each DS GelMA [0, 0.5%(w/v)].

### Evaluation of Hydrogel Elastic Moduli

One hundred microliters Hydrogels were pipetted into a 96-well plate and photo-cross-linked via UV exposure at 405 nm for 1 min. The hydrogels were swelled in PBS at 37°C overnight, and indentation was performed to determine elastic moduli. The stiffness of the hydrogels was characterized by nano-indenter (PIUMA, Optics11, Netherlands). For analysis, we selected an appropriate probe with Stiffness value = 0.5 N/m, Tip radius Value = 50 μm.

### Examination of Microstructure in Hydrogels

Hydrogels were frozen in liquid nitrogen, lyophilized and sputter coated with gold for 40 s and images were taken at 5 kV (PHENOM ProX SEM, Phenom-China). Porosity and pore sizes were calculated with Image J software (NIH). Each group had 3 hydrogel samples. Five images from each sample were randomly selected and 5 measurements from each image were taken.

### The Viability of Cells and Morphology on Hydrogels

The viability of cells in the hydrogels was assessed by cytotoxicity test of the extract. The extract of high density polyethylene [U.S. Pharmacopeial Convention (USP)] as the negative control and ZEDC polyurethanes (Tokyo Into industrial Co., Ltd.) as the positive control. The hydrogels were extracted aseptically, sealed and incubated in Vapor-bathing Constant temperature vibrator at 37°C and 60 rmp for 24 h. Fibroblast L929 cells were cultured in MEM medium at 37°C in a humidified atmosphere of 5% CO_2_, then digested by 0.25% trypsin. The suspended cells were dispensed at 100 μl per well in 96-well plate, and cultured in cell incubator (5%CO_2_, 37°C). After the cell grew to form a monolayer, original culture medium was discarded. The 96-well plates were then treated with 100 μl of extract of hydrogels, control article, negative article and positive article respectively. The 96-well plate was incubated at 37°C in cell incubator of 5% CO_2_ for 24 h. Then 50 μl aliquot of MTT (1 mg/ml) was added to each well and then incubated at 37°C for 2 h. The liquid in each well was tripped out and 100 μl isopropanol was added to each well to suspend the cell layer. The above suspension was evaluated with a dual-wavelength spectrophotometer with the measurement wavelength at 570 nm and reference wavelength at 650 nm.

Cell morphology was examined at 24 h culture on the surface of the hydrogels. Hydrogel polymerization was conducted in a 96 well plate (75 μl per well) and photo-cross-linked via UV exposure at 405 nm for 1 min. Then Mouse skeletal muscle cell line C2C12 single-cell suspension (1 x 10^5^ cells/ml) were dispensed at 100 μl per well in 96-well plate, and cultured in cell incubator (5%CO_2_, 37°C). After 24 h culture, C2C12 were fixed and stained with phalloidin (Beyotime) and DAPI to visualize F-actin filaments and cell nuclei respectively. Gels were washed in PBS and imaged on an OLYMPUS IX83 microscope.

### 3D Tumor Spheroid Formation and Invasion Study

Tumor cell cultures were trypsinized to generate a single-cell suspension and diluted to 200,000 cells/ml in growth medium. A volume of 50 μl single-cell suspension with tumor cells was added to each well of an anti-adhesion 96-well U-bottom plate (Corning, Corning, NY). The cells were pelleted into the u-bottom of each well by centrifugation at 500 × g for 5 min at room temperature using a centrifuge (Eppendorf, New York, NY). After centrifugation, 150 ml of growth medium was gently layered over each well. The plate was then incubated under standard culture conditions for 3 days.

The culture medium was removed and 50 μl of composite hydrogels (LGC, MGC, HGC) and Matrigel was added into each well. After polymerization, 150 μl of growth medium was gently layered over each well. The plate was then incubated under standard culture conditions. Olympus IX83 microscope photographs were taken on day 1, day 2, day 5, and day 7 to compare the tumor migration in composite hydrogels with Matrigel. Image analysis was performed with ImageJ to draw the spheroid boundary and measure the roughness and size of each cancer spheroid.

### Statistical Analysis

Data are presented as the mean ± standard deviation of at least three individual biological experiments. Comparisons between two groups were made by Student's two-tailed unpaired *T*-test. *P* < 0.05 was considered for the significance level for all analyses. Analysis of the data from NMR was performed using software MestReNova.

## Results and Discussion

### Determination of Degree of Methacrylation and Gelling Characteristics of GelMA

GelMA with varying degrees of methacrylation has previously been used to produce scaffolds with tunable mechanical properties (Benton et al., 2014). For instance, matrix stiffness could be changed via alterations to the methacrylation degree of GelMA. However, the effectiveness of GelMA containing composite hydrogels in modulating cancer cell growth and invasion have not been well-understood. In order to create an ideal *in vitro* micro-environment for cellular behavior studies, especially for cancer cells, we synthesized GelMA with MA at final concentrations of 3, 5, and 10% v/v. GelMA with low, medium, and high amount of MA modification are referred to as L-G, M-G, and H-G. The GelMA with different MA modification were confirmed with ^1^H NMR verification in correspond to acrylic protons (2H) of methacryloylated grafts of lysine groups (Figure 1B). These results demonstrated the ability to create GelMA polymers with a degree of methacrylation varying roughly from 20 to 95%. Three batches of GelMA were created with “high” (95 ± 0.2%), “medium” (65 ± 0.4%), and “low” (25 ± 0.6%) methacrylation degree.

The GelMA hydrogel polymerization was conducted when mixed with LAP (0.05% w/v final concentration) via UV exposure at 405 nm for 1 min. The transformation of GelMA from liquid to solid phase was demonstrated in Figure 1C. Cell spheroids, such as cancer spheroids, or iPSC-derived cardio-myocytes spheroids could be embedded in this gel perfectly while maintaining cellular functions (Figure 1D).

### Characterization of Composite Hydrogels

**Figure 2:**
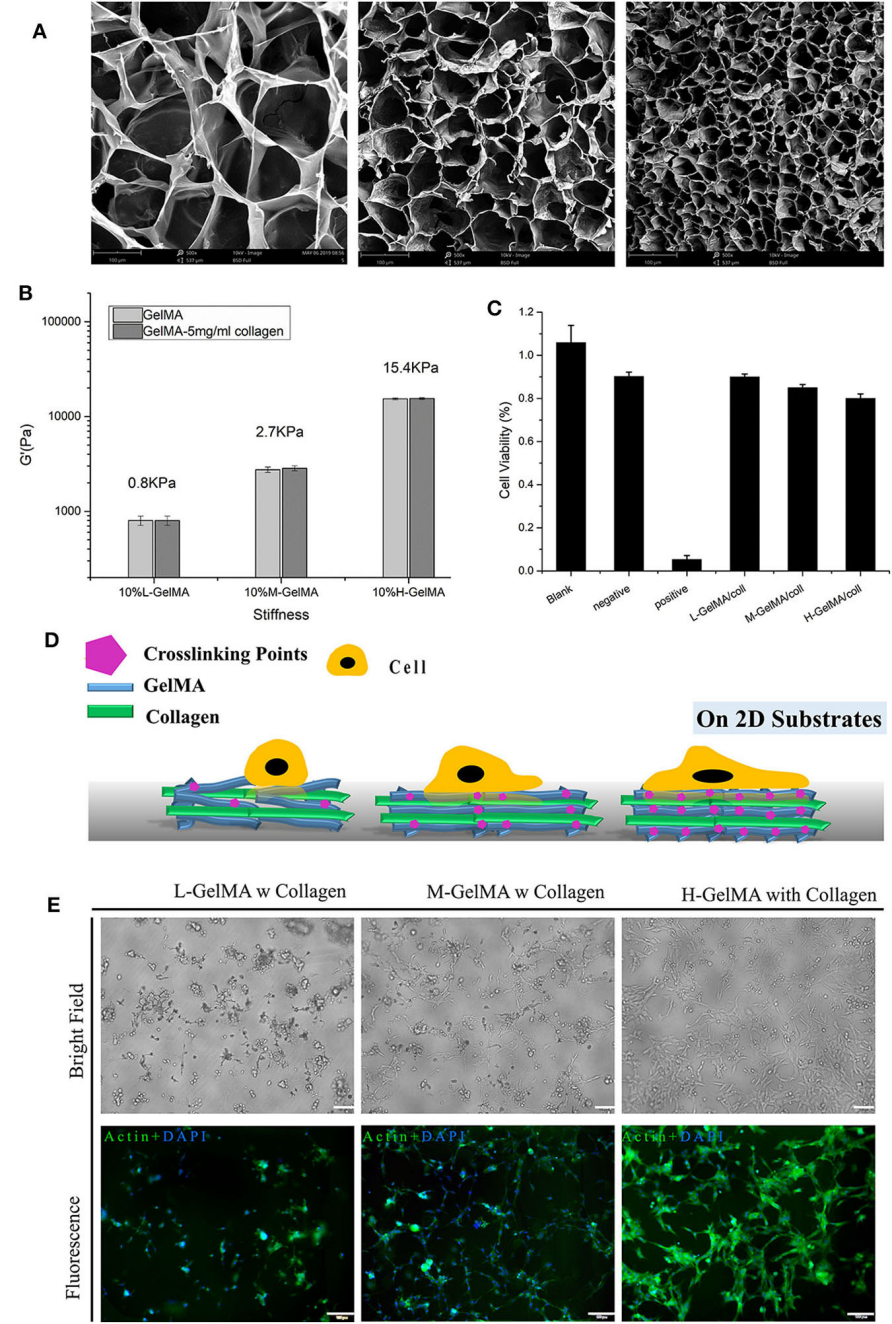
Mechanical properties of composite hydrogel. **(A)** SEM images of GelMA hydrogels, showing the effect of the degree of methacryloyl substitution on the ore sizes of GelMA hydrogels.; **(B)** three different degree of methacryloyl substitution of GelMA/collagen hydrogels were fabricated and collagen content was varied from 0 to 5 mg/ml, Despite variations in collagen content, under the same DS of GelMA, the stiffness of composite hydrogel was not significantly different. **(C)** Cell viability measured with different hydrogels, measured by MTT assay. **(D)** Schematic illustration of cell attachment on Composite hydrogel with different stiffness. **(E)** Morphology of C2C12 on 10% GelMA with different degree of substitution, I: L-G+C II: M-G+C III: H-G+C. *N* = 3 for each experiment. Error bars indicate the S.E.M of the data variation. Scale Bar, 100 μm.

To investigate how the changes in scaffold stiffness could affect cellular behavior, composite hydrogels with three different GelMA and collagen [0.5%(w/v)] were fabricated respectively. The porosity of the hydrogel was monitored with scanning electron microscopy (SEM) after lyophilization (Figure 2A). The SEM images revealed uniform porous microstructures throughout all the samples. SEM pictures show a homogenous microstructure with well-organized pores. The average size of interconnected pores decreased with higher degrees of methacrylation: 201.42 ± 5.87, 45.72 ± 7.12, and 16.45 ± 3.56 μm for the L-G, M-G, and H-G GelMA hydrogels, respectively. The mechanical properties of each synthesized GelMA hydrogel were evaluated using a nanoindenter tested. We selected the appropriate probe and test the elastic modulus of different methacrylation degree at GelMA concentrations of 10%(w/v). During the test, we performed mechanical testing in a water bath to avoid water loss, which can significantly affect the mechanical behavior of hydrogels (Data not shown). We found that the elastic modulus of the GelMA increased with its methacrylation degree: 142.56 ± 0.09 Pa (L-G), 3.75 ± 0.18 kPa (M-G), and 15.37 ± 0.33 kPa (H-G) (Figure 2B) and the concentration of collagen at 0.5%(w/v) did not influence the stiffness of the composite hydrogel. Collectively, the degree of methacrylation was found to affect the physical and mechanical properties of the synthesized GelMA hydrogels, with higher methacrylation resulting in stiffer and more durable hydrogels, with smaller pore sizes. To ensure the materials and polymerization conditions were not cytotoxic, Fibroblast L929 viability was measured when cultured with different material extracts. After 24 h, MTT assay was performed. The results showed that the cytotoxicity ratio of 100% test hydrogels extract was higher than 80% (Figure 2C). We observed acceptable viability for L929 cultured within the extracts of the composite hydrogel.

### Composite Hydrogel at Different Stiffness Led to Distinct Cell Behavior on 2D

Substrate stiffness plays an important role in the cellular adhesion formation and spreading (Figure 2D). Several previous studies have reported the regulation of cell behavior by substrates with different stiffness and the mechano-signaling involved (Discher et al., 2005, 2009; Han et al., 2018). Here, in order to test the cellular effect of stiffness change in composite hydrogel induced with different crosslinking, we used 2D cultured mouse skeletal muscle cell line C2C12 and placed them onto the hydrogel substrate. Results showed that on the softer substrate (L-G-C) (<1 kPa), majority of the 2D cultured C2C12 cells could not spread and attach firmly on the substrate; on a slightly stiffer substrate (M-G-C) (2.7 kPa), over 50% of cells could spread on the substrate and form spindle-like shape; on the hardest substrate (H-G-C) (15.4 kPa), majority of C2C12 cells could spread on the substrate entirely and formed a relatively large and flat cell morphology (Figure 2E). From the cell biology perspective, the above results demonstrated that substrates with different stiffness led to different cell-substrate adhesion patterns. These results also suggested that the composite hydrogel we made, could cover stiffness at a wide and defined range, which provided flexibility for researcher's usage. Considering that Matrigel is also a soft hydrogel (<1 kPa), the 3D organoids or cell spheroids could grow perfectly in the Matrigel system. The next question we asked, was whether or not the behavior of invasive and non-invasive tumors in the 3D environment, could be reconstructed and distinguished by our hydrogel series.

### Cancer Spheroids Behavior in 3D Composite Hydrogel

Matrigel based cell invasion analysis has been recognized as a “golden standard” for cancer invasion analysis (Benton et al., 2014). To explore if our composite hydrogel is close to the function of Matrigel in tumor invasiveness analysis, four kinds of tumor cells including invasive cells (MDA-MB-231 and NCI-H23) and non-invasive cells (MCF-7 and HT-29) were made to formed spheroids and embedded in our GelMA/collagen composite hydrogels, with comparison to pure Matrigel.

**Figure 3:**
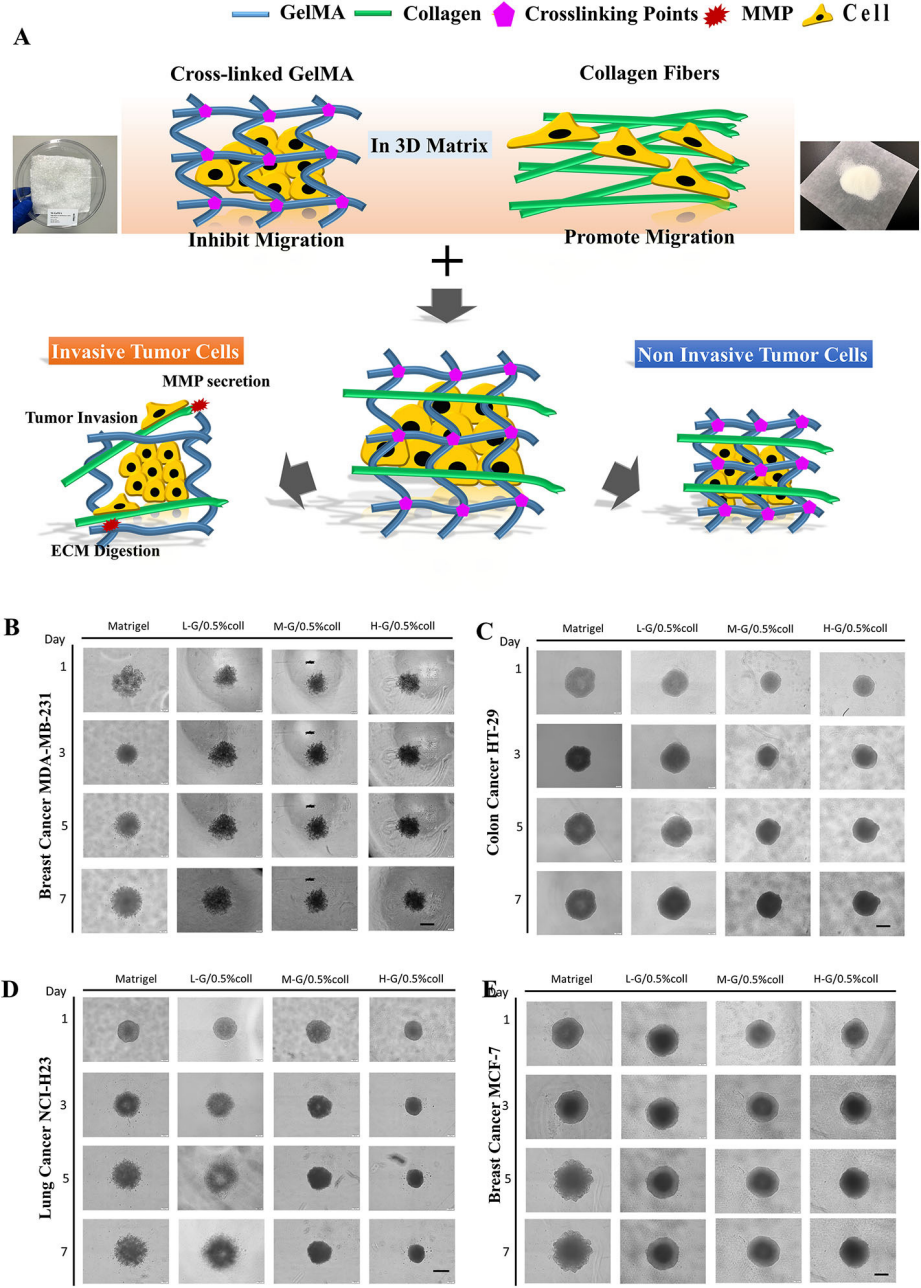
Tumor spheroid invasion in composite hydrogel. **(A)** Schematic illustration of Tumor invasion inside of hydrogels. GelMA hydrogel inhibits cell invasion while collagen promotes cell invasion. The composite hydrogel containing both GelMA and collagen component formed a 3D environment that allows invasive cells to digest matrix and invade, while the non-invasive cells were confined in their original positions. **(B–E)** are the comparison of cellular behavior among four types of cancer cells in Matrigel and in composite hydrogels, including: invasive breast cancer cells (MDA-MB231), less-invasive breast cancer cells (MCF7), invasive lung cancer cells (NCI-H23), and less-invasive colon cancer cells (HT29), respectively. Scale bar: 400 μm.

Bright-field microscope images were taken on day 1, day 3, day 5, and day 7 to compare the cancer spheroid morphology in different hydrogels. Images analysis was performed with ImageJ for spheroid size and boundary analysis. The size and the roughness of the spheroid were measured and compared to characterize the parameters for cancer growth and invasion. We found that the gel with higher degrees of methacrylation strongly inhibits the invasion of cells invasion (Figures 3B–E). The reason for that was the higher degree of methacrylation GelMA the higher stiffness of hydrogel, and the average size of interconnected pores were smaller. High stiffness and small pore diameter reduced the invasion of tumor cells (Thakuri et al., 2018).

Examination of the effect of increasing collagen density at each stiffness revealed that, across all stiffnesses, the addition of collagen was necessary for invasion to occur (Figure 3A). High collagen-containing hydrogel led to emerging of invasion behavior in non-invasive cancer cells (MCF-7 and HT-29), while high crosslinking GelMA led to significantly reduced cell invasion behavior in invasive cancer cells (MDA-MB-231 and NCI-H23). Collagen promotes the migration and invasion of cells since it has similar filamentous structure as natural ECMs, which plays a crucial role in tumor progression, invasion and metastasis. This property can control both the spatial organization of the cell-substrate adhesive ligands and mechanical signal transduction from cells to the collagen nanofibers. The hydrogel formed by highly-cross-linked GelMA can tightly wrap around the cells then impeded cell protrusion and migration. Therefore, in order to simulate the Matrigel function in cancer invasion studies, there is a delicate balance between these two components—a suitable concentration of collagen and GelMA in the composite hydrogel is required.

**Figure 4:**
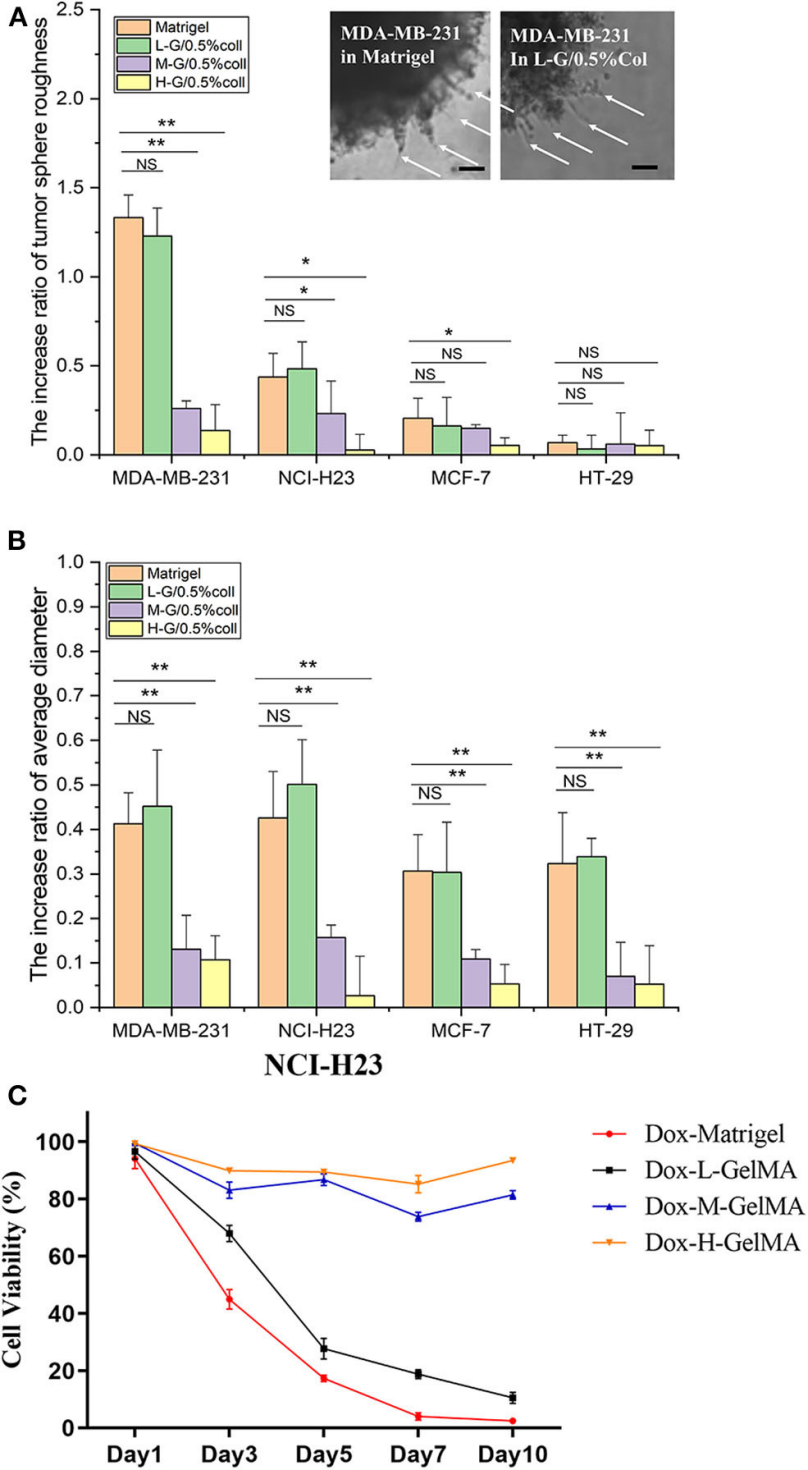
Quantitative analysis of tumor spheroid behavior in composite hydrogel. **(A,B)** The spheroid roughness and size comparison of four kinds of tumor microspheres in Matrigel and composite hydrogel. Inserted image shows the invadopodia-like protrusions inside of the both Matrigel and L-G-C composite hydrogel. **(C)** The drug efficacy to cancer cells tested in Matrigel and L-G-C composite hydrogel. *N* = 3 for each experiment. Error bars indicate the S.E.M of the data variation. Significance were indicated with *P < 0.05, **P < 0.01 (one way ANNOVA with Tukey back test). Scale bar: 50 μm.

Matrigel is recognized as a “golden standard” scaffold has several notable limitations for tissue engineering as we summarized above. These include, first, the variability of inherent composition usually results to uncontrollable individual specific microenvironments. Second, the differences between batch-to-batch and unmanageable degradation make it difficult to distinguish the true ECM component which is responsible for the cell fate, as well as to reproduce the comparison of cell growth in cancer cell studies. The fast gelling of Matrigel and lacking of consistency can result problems during cell culture. In our study, we developed a novel method for fabricating GelMA-based hydrogels to simulate the Matrigel for culture tumor spheroids. 5% L-G GelMA with 0.5% collagen could be used as basic scaffold. The invasion of invasive cells—MDA-MB-231 and NCI-H23—in the GelMA-based hydrogel (5% L-G GelMA with 0.5% collagen) were almost the same as in the Matrigel, judging from the cell morphology in the gel—the invadopodia/filopodia like protrusions (Figure 4A inserted images)—and by the comparison of size and roughness of the cancer spheroid (Figures 4A,B). The invasion and growth state of non-invasive cells (MCF-7 and HT-29) were also quite similar to as they were in the Matrigel. As a result, we concluded this recipe of the composite gels could be an alternative to or a replacement of Matrigel for tumor spheroids culture and invasion analysis.

### The Application of the Composite Hydrogel in 3D Bioprinting and Creating Channels and Structures for Tumor-on-a-Chip Research

**Figure 5:**
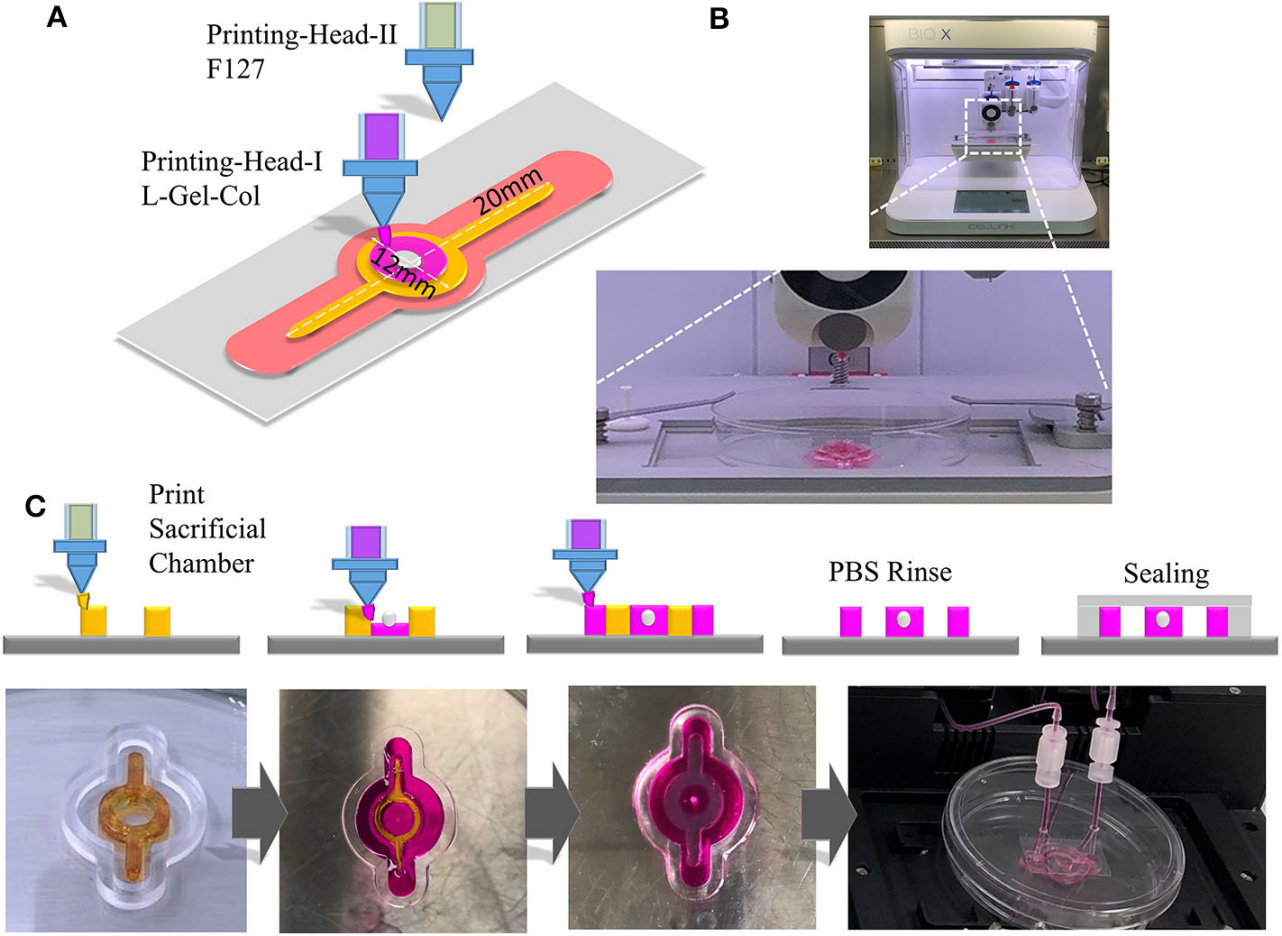
3D Printing with composite hydrogel and fabrication of cancer spheroid containing Tumor-on-a-Chip. **(A)** Schematic illustration of 3D printing of a cancer spheroid containing flow chamber for cancer invasion and metastasis study. **(B)** Chip in 3D printing with BioX bio-printer (CellInk). **(C)** Upper panels are the schematic illustrate of the processing of the flow chip. Chip was first printed with F127 as a sacrificial layer. Then the L-G-C was printed and photo-crosslinked in the center of chip with cancer spheroids embedded, and the outer layer of the chip. The F127 layer was dissolved with phosphate buffer forming hollow channels for perfusion and the glass sealing was performed. Lower panels are the actual photo for the printed chip at each steps.

**Figure 6:**
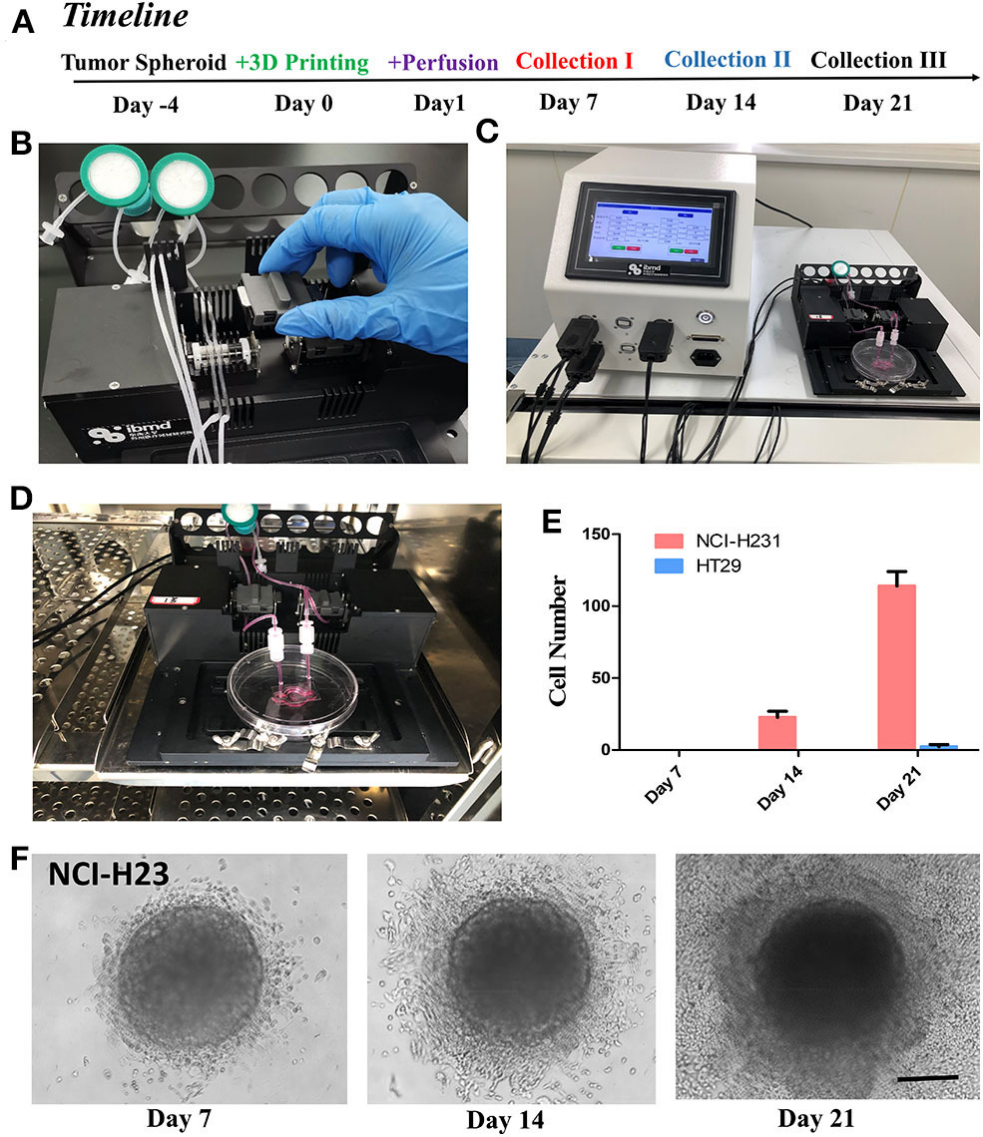
Perfusion of the tumor-on-a-chip and study of cancer invasion and metastasis. **(A)** The timeline of the experiment with the cancer spheroid containing flow microchip for cancer invasion and metastasis study. **(B)** The setup of peristatic pumping system. Each chip has two channels for inlet and out perfusion. **(C)** The controller of the perfusion system, allowed automated perfusion of chip. **(D)** The microchip and entire circulation system were setup in incubator, and perfused at 0.2 ml/min for up to 21 days with three times of sampling procedures and medium changes. **(E)** Cell metastasized and harvested from cancer spheroids from the perfusion medium on day 7, 14, and 21. **(F)** NCI-H23 cell in the L-G-C composite hydrogel with in Day 7, 14, and 21. Cells had distinguished invasion into the 3D-Matrix and migrated out of the gel into the micro-channels. *N* = 3 for each experiment. Error bars indicate the S.E.M of the data variation. Scale bar: 200 μm.

GelMA is a bioprinting material (Yin et al., 2018). Since GelMA is one of the main components of our composite hydrogel, we explored the usage of this biomaterial for bio-3D printing, to form and obtain the desired microstructure and corresponding microfluidics. In order to test the printing characteristics of the material, we designed a Tumor-on-a-Chip with a core containing 3D tumor spheroids and circular flow channels around the tumor spheroids (Figure 5A), and used BioX printer (Cellink) (Figure 5B) to print and construct the chip. The process of the printing was shown in Figure 5C. We first printed the sacrificial layer with F127 to split the channels, and then printed L-G-C hydrogel to form the core part of the chip—the tumor spheroid made by invasive NCI-H23 cells or low invasive HT29 cells—and the external channel wall of the chip. After printing, we dissolved the F127 sacrificial layer with PBS solution to generate the channels all through the chip. Figure 5C is the schematic illustration and real images of the bioprinting in each step. After that, the chip was packaged with the outer bracket having a PMMA chamber structure obtained by machining, and the bottom having a glass slide. The inlet and outlet were positioned on the top of the chip and sealed with a screw and an O ring. The timeline of the cancer flow chamber experiment was shown in Figure 6A. We have prepared customized perfusion system and fluid control device for this experiment. The perfusion system uses the peristaltic pump (LONGER) installed on the self-made bracket for chip perfusion (Figure 6B), and the perfusion speed was set at 0.2 ml/min. The perfusion system also includes a medium reservoir (15 ml) and an 0.22 μm air filter. The control device had a touchable LED screen and corresponding software to setup perfusion speed and time, which could be freely programed (Figure 6C). The perfusion system was placed inside the incubator for long-term cell cultivation (Figure 6D). The medium of the systems was changed twice a week, and every 7 days, a 2-h continuous fluid perfusion was performed to collect direct flow through medium, in which the circulating tumor cells were harvested and analyzed. From the experimental results, the invasive NCI-H23 lung cancer cells can invade the circulatory system in about 14 days, and a large number of invasive cells can be detected in the circulating fluid of the chip after 21 days. Compared with the low invasive tumor cells, no cells were detected in the circulating flow path at 14 days, and only a few exfoliated cells were detected at 21 days (Figures 6E,F).

### The Potential of the Composite Hydrogel as a Substitute for Cancer Spheroid Culture and Invasiveness Evaluation

Finding a chemically defined, multi-parameters adjustable—such as stiffness and porosity—and inexpensive novel hydrogels to replace Matrigel, is currently the focus of biomaterials research (Kratochvil et al., 2019). The most critical criterion for this novel hydrogel is its regulation of three-dimensional tumor growth and invasion, the invasive tumor in Matrigel is also invasive in the new gel, meanwhile the non-invasive tumor in Matrigel behave non-invasive in the new gel. In this paper, we used the mixture of GelMA at different degree of MA substitution and collagen altogether and fabricated a composite hydrogel. We had surprisingly positive results in regulating its porosity (Figure 2A) and hardness (Figure 2B), together with the tests of 3D tumor invasion abilities (Figures 3, 4). This depends on the joint action of the positively regulatory component—Collagen—that promotes cell invasiveness, and the negatively regulatory component—GelMA—that inhibits cell migration (Figure 3A). In testing of invasion of four representative tumor cells, we found that there was a good correspondence between their migration in LGC gel as to their migration in Matrigel (Figure 4). Then we tested the drug effect on the cancer spheroid between Matrigel and composite hydrogels, we found that the trend of the drug effect of LGC hydrogel was similar to the Matrigel. This enables the LGC hydrogel to have a good potential to replace Matrigel for the tumor three-dimensional migration studies. The application of the LGC gel in 3D printing and Tumor-on-a-Chip based tumor invasive detection further strengthen its advantages over Matrigel.

There are some deficiencies in this work. In order to confirm that this a material that can be used to completely replace Matrigel for tumor growth and invasion research. More tumor tests are required, including studies with primary tumors from different sources and tumor organoids culture—as the current trend in tumor cell biology research. The tumor invadopodia, filopodia, lobopodia, and other parameters could be systematically analyzed and compared to results from Matrigel in a mapping table format (Yamada and Sixt, 2019). This part of work will be performed in our future research.

## Conclusions

In this paper, we developed a composite hydrogel with adjustable stiffness and porosity from mixation of GelMA, crosslinker, and collagen. We demonstrated this hydrogel had similar properties as Matrigel in 3D tumor spheroids culture and tumor invasiveness studies. This LGC hydrogel was further successfully applied in 3D-bioprinting and constructed a Tumor-on-a-Chip for cancer invasion and metastasis studies. Our research demonstrated this hydrogel has multiple advantages thus have a good potential to replace Matrigel for the tumor three-dimensional invasion studies.

## Data Availability Statement

The original contributions presented in the study are included in the article/supplementary materials, further inquiries can be directed to the corresponding author/s.

## Author Contributions

ZC, FW, JZ, XS, YW, and JO performed all of the experiments. ZC, FW, TH, and ZG designed the project and wrote the manuscript. JZ, YY, and JG developed and produced hardware and software of perfusion controlling system. JZ, TH, and ZG helped for manuscript editing and data analysis. All authors contributed to the article and approved the submitted version.

## Conflict of Interest

The authors declare that the research was conducted in the absence of any commercial or financial relationships that could be construed as a potential conflict of interest.
